# Use of a platelet-rich plasma-collagen scaffold as a bioenhanced repair treatment for management of partial cruciate ligament rupture in dogs

**DOI:** 10.1371/journal.pone.0197204

**Published:** 2018-06-19

**Authors:** Susannah J. Sample, Molly A. Racette, Eric C. Hans, Nicola J. Volstad, Susan L. Schaefer, Jason A. Bleedorn, Jeffrey P. Little, Kenneth R. Waller, Zhengling Hao, Walter F. Block, Peter Muir

**Affiliations:** 1 Comparative Orthopaedic Research Laboratory, Department of Surgical Sciences, School of Veterinary Medicine, University of Wisconsin-Madison, Madison, Wisconsin, United States of America; 2 Department of Medical Physics, School of Medicine and Public Health, University of Wisconsin-Madison, Madison, Wisconsin, United States of America; Mayo Clinic Minnesota, UNITED STATES

## Abstract

Dogs are commonly affected with cruciate ligament rupture (CR) and associated osteoarthritis (OA), and frequently develop a second contralateral CR. Platelet rich plasma (PRP) is a component of whole blood that contains numerous growth factors, which in combination with a collagen scaffold may act to promote bioenhanced primary repair of ligament. This study tested the hypothesis that treatment of partial stable CR stifles with an intra-articular collagen scaffold and PRP would decrease the disease progression, synovitis and risk of complete CR over a 12-month study period. We conducted a prospective cohort study of 29 client-owned dogs with an unstable stifle due to complete CR and stable contralateral stifle with partial CR. All dogs were treated with tibial plateau leveling osteotomy (TPLO) on the unstable stifle and a single intra-articular application of PRP-collagen in the stable partial CR stifle. Dogs were evaluated at the time of diagnosis, and at 10-weeks and 12-months after treatment. We evaluated correlation between both development of complete CR and time to complete CR with diagnostic tests including bilateral stifle radiographs, 3.0 Tesla magnetic resonance (MR) imaging, and bilateral stifle arthroscopy. Additionally, histologic evaluation of synovial biopsies, C-reactive protein (CRP) concentrations in serum and synovial fluid, and synovial total nucleated cell count, were determined. Results indicated that a single application of PRP-collagen in partial CR stifles of client owned dogs is not an effective disease-modifying therapy for the prevention of progression to complete CR. Radiographic effusion, arthroscopic evaluation of cranial cruciate ligament (CrCL) damage, and MR assessment of ligament fiber tearing in partial CR stifles correlated with progression to complete CR over the 12-month follow-up period. We determined that the best predictive model for development of complete CR in PRP-collagen treated partial CR stifles included variables from multiple diagnostic modalities.

## Introduction

Cruciate ligament rupture (CR) is a common naturally occurring degenerative condition of the canine stifle. It is an economically important disabling disease that causes ~20% of lameness in dogs [1]. CR is a complex disease with moderate heritability [2]. Bilateral CR occurs in up to 60% of affected dogs [1,3]. Ligament sprains should be defined biomechanically [4]. Grade I sprains have mild fiber damage and no associated joint laxity. Grade II ligament sprains have moderate fiber damage such that joint laxity is just appearing due to damage-induced loading. Grade III ligament sprains have severe disruption of ligament fibers and joint laxity that is obvious [4]. Therefore, Grade III CR stifle joints are unstable clinically. Stifles with partial CR are clinically stable with Grade I sprains with associated ligament fiber rupture evident using arthroscopic examination [5].

During development of CR, stifle osteoarthritis (OA) and ligament fiber damage progressively affect both the cranial cruciate ligament (CrCL) and the caudal cruciate ligament (CaCL). In the early phase of the disease, affected stifles with partial CR are clinically stable, but affected dogs are often lame [6]. Palpable periarticular fibrosis may be clinically found in stifles with partial CR [1,3,5]. Radiographic signs of synovial effusion and OA are often present in partial CR stifles, and are important signs that support the diagnosis of partial CR [1]. Severity of radiographic change is predictive of risk of complete CR [1,3,7]. On arthroscopic examination of partial CR stifles, ligament fiber rupture is evident [5]. On histologic examination of tissue from dogs with CR, ligament matrix degeneration, loss of ligament fibroblasts, fiber rupture, and synovitis are typical [5,8].

Dogs are diagnosed with CR most commonly in the late phase of the disease, when sufficient ligament fiber damage has caused mid-substance rupture of the CrCL, resulting in stifle instability without contact trauma [1,7]. Dogs with complete CR typically have more severe lameness and are at high risk of developing secondary meniscal damage. Fiber damage to the CaCL is common in affected dogs [9], but complete rupture of both the CrCL and CaCL is uncommon. Lymphocytic-plasmacytic synovitis and associated radiographic change is typical in affected unstable joints [1,5,10,11]. Severity of histological synovitis in complete CR stifles is predictive of risk for subsequent contralateral complete CR [12]. Management often includes surgical stabilization of the stifle without ligament repair. Tibial plateau leveling osteotomy (TPLO) stabilization is most strongly supported by clinical outcomes [13]. Surgical stabilization is not disease-modifying; synovial effusion and stifle OA typically progress over time when unstable CR stifles are treated with TPLO [14]. Currently, there is no disease-modifying treatment that is known to alter progression of CR in dogs.

Neither the pathologic processes underlying initiation of CR nor the cause of progression from partial CR to complete CR are fully understood. Synovitis and OA are components of early phase disease in stifles with partial CR [5]. Given that induction of immune synovitis in animal models results in a reduction in CrCL strength and eventually leads to mid-substance rupture [15], the inflammatory environment in stifles with partial CR is likely an important component in the pathogenesis of progressive ligament rupture.

The intra-articular, extra-synovial location of the cruciate ligaments impairs healing [16]. For example, synovial plasmin impairs formation of the fibrin-platelet plug necessary for healing [16], and lack of a provisional scaffold at the injury site inhibits ligament fibroblast proliferation and collagen synthesis [17]. The lack of a sufficient provisional scaffold to enable damaged cruciate ligament to heal has led to investigations into bioengineered scaffolds suitable for an intra-articular environment. Cruciate ligament healing can be augmented by the use of a collagen scaffold and a source of growth factors, such as platelet rich plasma (PRP), as a bioenhanced primary repair [18]. PRP is a component of whole blood that is processed to obtain a plasma concentrate with 3 to 5 times more platelets than normal blood. Platelets are an integral part of wound healing and represent a potentially valuable source of growth factors. PRP contains numerous growth factors, including fibroblast growth factor-2 (FGF-2), transforming growth factor β-1 (TGF-β1), insulin-like growth factor (IGF-1), platelet-derived growth factor (PDGF) and vascular endothelial growth factor (VEGF), which have been shown in both *in vivo* and *in vitro* models to enhance cell proliferation, cell migration, angiogenesis and extracellular matrix production in numerous cell types including human anterior cruciate ligament (ACL) fibroblasts [19–22]. Prior work evaluating use of PRP to bridge the gap in a transected ACL was not shown to enhance healing [23], while addition of PRP to a collagen hydrogel scaffold increased metabolic activity, stimulated collagen production and reduced apoptotic rate in a pig model [24]. Use of a PRP-collagen hydrogel in a naturally occurring canine model of partial CR has not been investigated.

The present study aimed to evaluate use of a PRP-collagen hydrogel as a means to attenuate synovitis and prevent progressive cruciate ligament fiber rupture in dogs. We used an established naturally occurring CR disease model [1,7,25] and studied a cohort of dogs with unilateral complete CR and contralateral partial CR [5,25]. We hypothesized that treatment of partial CR stifles with an intra-articular collagen scaffold and PRP would decrease the disease progression, synovitis and risk of complete CR over a 1-year follow-up period.

## Materials and methods

### Dogs

**Fig 1:**
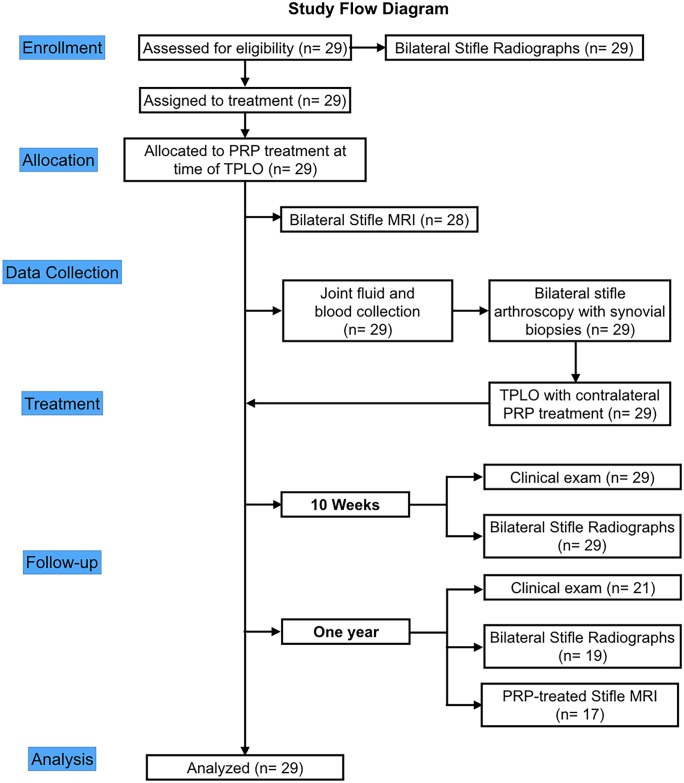
Study design flow diagram illustrating enrollment, data collection, treatment and analysis. PRP—platelet rich plasma; TPLO—tibial plateau leveling osteotomy for treatment of stifle instability from CR; MRI—magnetic resonance imaging.

Twenty-nine with unilateral complete CR and contralateral partial CR were enrolled in this study. These dogs were also used in a prior study [25], for which the inclusion and exclusion criteria described here were used. Dogs were prospectively recruited to the study at the University of Wisconsin-Madison UW Veterinary Care Hospital between April 2013 and June 2014 (Fig 1). To qualify for this study, dogs had to present with clinical signs of unilateral pelvic limb lameness due to complete CR as determined by palpable cranial tibial translation, and have radiographic evidence of bilateral stifle synovial effusion and osteophytosis [1]. Dogs were excluded if there was evidence of Grade II or III sprain with obvious joint laxity in the contralateral stifle under sedation, if examination and history suggested traumatic injury, if previous stifle surgery had been performed, or if other stifle pathology not associated with the CR condition was present. Age, weight, body condition score, gender, and history were recorded for each dog. Cranial drawer and cranial tibial thrust tests were used to assess passive stifle laxity in flexion and extension under sedation [26] and repeated under general anesthesia. After diagnosis, the complete CR stifle was treated using TPLO, while the contralateral partial CR stifle was treated with a PRP-collagen scaffold. Owners were sent home with specific instructions regarding post-operative activity, including strict rest for the initial 2 weeks after surgery followed by gradual increases in low impact activities for the subsequent 8 weeks until recheck evaluation. At the time of discharge, owners and dogs had a physical rehabilitation appointment, during which owners were trained in manual therapy, modalities for swelling and pain management and gait training with a sling; additional therapies were determined for each individual patient. Dogs were evaluated at 10 weeks and 12 months after treatment. In cases where the PRP-collagen treated stifle developed complete CR rupture within the 1-year study period, the time to rupture was recorded in days.

### Ethics statement

All procedures were conducted with the approval of the Animal Care & Use Committee, School of Veterinary Medicine, University of Wisconsin-Madison (V1070). Dogs were recruited with informed written consent from each owner.

### PRP-collagen application

Collagen slurries were prepared as previously described [27]. A standard PRP isolation method was used to prepare the PRP shortly before surgery (SmartPReP^®^2 APC^+™^ Harvest Technologies, Plymouth, MA). Briefly, at the time of anesthetic induction, 2mL of blood was drawn for a platelet count, and 32mL of blood was collected in a PRP isolation syringe, which underwent centrifugation to produce PRP. Platelet counts from the whole blood and PRP were determined using an automated hematology analyzer. The intercondylar notch of the partial CR stifle was approached using a medial parapatellar mini-arthrotomy. After arthroscopic examination and synovial biopsy collection was completed, PRP was added to the collagen slurry at a 1:1 ratio. The intercondylar notch was filled with approximately 2mL of the PRP-collagen mixture that formed a hydrogel within a few minutes. After the PRP-collagen was determined to be the consistency of a soft gel, the arthrotomy was closed.

Platelet counts were used to determine percent platelet recovery using the equation: Percentage platelets recovered = [(Nr x Vr)/(Nt x Vt)] x 100, where Nr is the platelet concentration from the PRP, Vr is the total volume of PRP produced after centrifugation, Nt is the platelet concentration of the whole blood, and Vt is the total blood volume collected. The platelet concentration factor of platelets within the PRP was also determined and recorded for each dog, using the equation: platelet concentration factor = Nr/Nt.

### Radiography

Weight-bearing lateral medial stifle radiographs were made bilaterally at the time of diagnosis, at the 10-week recheck and at the 12-month recheck; only dogs that did not develop a second complete CR had 12-month recheck radiographs. Weight-bearing radiographs were obtained to determine the functional length of the CrCL under load [28]. Orthogonal cranial caudal and medial lateral radiographic views of both stifles were also obtained at the time of diagnosis under sedation with the stifle flexed to ninety degrees. Radiographs from both stifles were evaluated at all time points for effusion, OA, and the functional length of the CrCL. Tibial plateau angle (TPA) was measured for both stifles using radiographs obtained at the time of diagnosis. Radiographic effusion and radiographic OA were used to infer severity of stifle synovitis [1]; the most important early radiographic sign of stifle OA in dogs with CR is development of synovial effusion and associated compression of the infrapatellar fat pad [1,3]. Radiographic effusion was graded subjectively on a scale from 0–2 (0—normal, 1—mild, 2—severe) [1], for which cranial and caudal stifle joint spaces were considered. Radiographic OA was graded subjectively on a scale from 0–3 (0—normal, 1—mild, 2—moderate, 3—severe) based on the severity of osteophytosis at the margins of the stifle joint [1]. TPA was measured from lateral radiographic views of the tibia obtained with the stifle and tarsus held in ninety degrees of flexion. Using weight-bearing radiographs, CrCL functional length was measured as the distance between the femoral and tibial attachments [28], and normalized to patellar length (CrCL_d_). Radiographic scoring was performed by a single observer (PM). To enable comparison of development of complete CR with respect to radiographic effusion and OA in partial CR stifles at diagnosis, published control data from the author’s laboratory [1] was used, which included 83 dogs with the same inclusion criteria as described above, although these dogs did not have disease-modifying therapy applied to the partial CR stifle.

### Magnetic resonance imaging

Magnetic resonance (MR) imaging of both stifles was performed at diagnosis. Follow-up MR imaging of the PRP-collagen treated stifles were obtained from dogs that did not progress to complete CR by the 12-month recheck. All MR imaging sequences were obtained under general anesthesia. For general anesthesia, dogs were pre-medicated with dexmedetomidine (2-4mcg, intramuscular (IM)) and hydromorphone (0.1–0.2mg/kg IM), induced with propofol (2-10mg/kg) and maintained using isoflurane. A 3.0 Tesla MR imaging scanner (GE 3T 750 Discovery, GE Healthcare, Waukesha, WI) was used. 3D fast spin echo (FSE) Cube sequences were obtained for all imaged stifles. For PRP-collagen treated stifles, Vastly under-sampled Isotropic PRojection with alternating length repetition times (VIPR-aTR) sequences, and pre- and post-contrast (intravenous gadolinium, 50–60 mg/kg, MultiHance, Bracco Diagnostics Inc, NJ) sagittal T1-weighted sequences, were also obtained. MR sequence specifications for the 3D FSE Cube were TR—1600ms, TE—22.0ms, flip angle—90 degrees, and an image matrix size of 384 x 384 x 60 over a 140 x 140 x 60mm field-of-view for a resolution (voxel dimension) of 0.36 x 36 x 1mm. For the VIPR-aTR sequence, specifications were TR—4.6ms and flip angle—15 degrees. Two radial lines are acquired per TR in an out and back trajectory over 3.6ms echo times of 0.5 and 3.2ms. VIPR-aTR has an image matrix size of 320 x 320 x 320 over a 120 x 120 x 120mm field-of-view for a 0.375 x 0.375 x 0.375mm voxel dimension. MR sequence specifications for the T1-weighted sequence were TR—700ms, TE—10.9ms, flip angle—90–111 degrees, and an image matrix size of 384 x 224mm over a 160 x 160mm field-of-view for a resolution (voxel dimension) of 0.41 x 0.71mm.

Sequences were chosen and oriented to optimize cruciate ligament imaging. 3D segmentation software (Mimics, Materialise, Belgium) was used to measure CrCL volume and median grayscale value in the 3D FSE Cube and VIPR-aTR sequences. 3D FSE Cube and VIPR-aTR CrCL volume measurements were normalized to radiographic patella length (CrCL FSE Volume, CrCL VIPR Volume, respectively) [25]. Median CrCL grayscale values for the 3D FSE Cube and VIPR-aTR sequences were normalized to the grayscale value of the cranial tibial muscle (CrCL FSE Grayscale, CrCL VIPR Grayscale, respectively); the cranial tibial muscle was selected for normalization because its large volume in the image field would minimize background signal variation [25,29]. To estimate CrCL ligament contrast enhancement (CrCL T1 Enhance), the difference between the median grayscale values before and after contrast injection, both of which were normalized to the cranial tibial muscle median grayscale value pre-contrast, was determined from T1-weighted sequences [25]. Volume, grayscale and T1 image measurements were made by a single observer (NV). Using 3D FSE Cube sagittal plane images of the intercondylar notch, CrCL fiber loss was evaluated using a visual analog scale (0–100), with 0 representing no fiber loss, and 100 signifying complete ligament fiber loss (CrCL fiber loss). Criteria for CrCL fiber loss included change in CrCL volume, changes in CrCL signal intensity and ligament discontinuity [30]. Fiber loss measurements were made by a single observer (SLS).

### Arthroscopy

Before TPLO, both stifles were examined through a medial parapatellar mini-arthrotomy incision using a 2.7 or 2.9 mm 30° rigid arthroscope. Procedures occurred under general anesthesia, which was induced with propofol (2-10mg/kg) and maintained with isofluorane; the specific pre-anesthetic medications used varied between dogs and were based on individual clinical assessment and the discretion of the supervising anesthesiologist. Joint regions (lateral and medial pouches, lateral and medial femoro-tibial joint compartments, the intercondylar notch, and the femoro-patellar joint) were evaluated [10,25]. Severity of synovitis and cruciate ligament fiber damage were graded using a standardized scoring system [10,25]. Three parameters describing macroscopic inflammation were evaluated for each joint region: synovial hypertrophy, vascularity, and synovitis [10,25]. A total arthroscopic synovitis score was calculated as the sum of each parameter’s grade [25,31]. Additionally, the degree of global joint synovitis was scored using a visual analog scale (VAS) (0–100) for each stifle joint, with 0 representing no inflammation, and 100 signifying the most severe inflammation (Synovitis VAS) [10,25]. The CrCL and the CaCL were inspected and probed for evidence of pathologic change, including fiber rupture, color change, and hypertrophy of ruptured ends of ligament fascicles. The extent of fiber disruption was estimated using a calibrated arthroscopic hook probe. The degree of CrCL fiber tearing in the partial CR stifle (CrCL Fiber Damage VAS) was determined using a visual analog scale (0–100), with 0 representing no damage, and 100 signifying complete ligament fiber rupture [25]. The lateral and medial menisci were also inspected and probed for tears, which were treated by resection if present. A biopsy of synovial membrane was collected from the medial joint pouch of each stifle after completion of arthroscopic observation. For the partial CR stifle, PRP-collagen was applied to the intercondylar notch as described above before joint closure.

### Histology and histomorphometry

Synovial biopsies from the medial compartment of both stifle joints were collected during arthroscopic examination, and immediately fixed in Zamboni’s fixative for 1–2 days at 4°C [32]. Multiple 10μm thick paraffin-embedded sections were prepared. Sections were stained with hematoxylin and eosin (H&E) stain for assessment of cellular infiltration. Lymphocytic-plasmacytic and suppurative inflammation of the synovial intima, synovial cell hypertrophy, and synovial intima width, seen at the center of five random high power fields, were subjectively graded by a single grader (PM) using numerical scales [25,31]. Lymphocytic-plasmacytic inflammation was graded from 0 to 3 using the following scale: 0—no inflammation, 1—infiltration without alteration of tissue architecture, mostly perivascular or sub-intimal, 2—infiltration with occasional alteration of tissue architecture, numerous inflammatory cells, 3—infiltration with extensively obscured tissue architecture, dense inflammatory infiltrate and/or follicle formation. Suppurative inflammation was graded as: 0—no inflammation, 1—negligible inflammation; 2—present. Synoviocyte cell hypertrophy was graded as follows: 0—normal, 1—mild, 2—moderate or focal severe, 3—severe. Synovial lining cell layer width, seen at the center of five random high power fields, was measured to determine lining layer depth of each biopsy sample [33] and graded as follows: 0—one cell layer, 1—2–3 cell layers, 2—4–5 cell layers, 3—>5 cell layers. A synovial pathology score for each stifle was calculated as the sum of the scores assigned to lymphocytic-plasmacytic inflammation, synoviocyte cell hypertrophy and synovial line cell layer thickness. A synovial pathology grade (Synovitis Grade) was assigned based on the synovial score (grade 0/no synovitis = sum 0–1; grade 1/low-grade synovitis = sum 2–4; grade 2/high-grade synovitis = sum 5–9) [31]. Additionally, a visual analog scale score for overall synovitis (Synovial Inflammation VAS) was determined using a 100mm horizontal line, the end-points of which were set by the descriptor of no inflammation (0) and most severe inflammation (100).

### Synovial and serum markers of inflammation and ligament matrix degradation

Stifle synovial fluid and blood were collected at the time of diagnosis, and at the 10-week and 12-month rechecks; only dogs that did not develop a second complete CR had 12-month synovial fluid and blood samples collected. Synovial fluid total nuclear cell count (TNCC) in TPLO-treated and the PRP-collagen treated stifles was estimated using direct smears of synovial fluid obtained by arthrocentesis and stained with Wright-Giemsa stain. Quality control for direct smears was as described, and manual counting of synovial cells on each smear was performed using a validated estimation method [34]. All nucleated cells in a field were counted, including those with pyknotic and karyorrhectic nuclei, cells forming groups, and naked nuclei. A cell was considered within the counting field if at least half the cell was within the microscopic field. Nucleated cells in fifteen 400x fields were counted and the mean number of nucleated cells per 400x field was determined. After correction for the dimensions of the 400x field area, TNCC was estimated (TNCC) using the regression formula (y = 0.45x-0.36). C-reactive protein (CRP) was quantified in serum and synovial fluid (Serum CRP, Synovial CRP) using commercial canine-specific ELISAs (Immunology Consultants Laboratory, Ict., Portland, OR). Synovial fluid/serum CRP ratios were also calculated (Synovial:Serum CRP).

### Statistical analysis

Variables used for analysis, when they were collected, and number of dogs for which each variable obtained is summarized in S1 Table. The Shapiro-Wilk test was used to access normality for all variables. Data were reported as mean ± standard error or median (range) as appropriate. The Spearman Rank correlation test was used to determine correlation between variables obtained at diagnosis and both time to, and occurrence of, complete CR in PRP-collagen treated stifles over the 12-month study period. Cox’s proportional hazard model was used to formulate a predictive model of time to complete CR in the PRP treated stifles. Variables included in this model were determined by undertaking univariate Cox’s regression on all individual variables obtained at the time of diagnosis and choosing unrelated variables at *P*<0.2 for further analysis (S3 Table). An additional model was also formulated using only a subset of variables that could be easily obtained radiographically. The Wilcoxon matched-pairs sign rank test was used to compare individual variables obtained at two time points. The Friedman test, with a Dunn’s post-hoc test when appropriate, was used to compare individual variables obtained at three time points. For evaluation of radiographic measures for which historic control data is available [1], the Chi-squared test was used to evaluate differences in 12-month rupture rates between partial CR stifles and PRP-collagen treated partial CR stifles. Kaplan-Meier curves describing time to complete rupture for partial CR stifles and PRP-collagen treated partial CR stifles were also compared using the Log-rank (Mantel-Cox) test [1].

## Results

### Clinical findings

Twenty-nine dogs, aged 5.5±0.5 years (range: 1.6 to 9.9 years), were prospectively enrolled (Fig 1). Body weight was 37.1±1.7kg (range: 24.1 to 59.0kg). There were 14 ovariohysterectomized females, 3 males, and 12 castrated males. A range of breeds was represented, including 11 Labrador Retrievers, 6 mixed breed dogs, 3 Golden Retrievers, 2 Boxers, and one each of the following: Dalmatian, Springer Spaniel, German Shepherd Dog, German Shorthair Pointer, French Mastiff, Dalmatian, Springer Spaniel, Rottweiler, and Newfoundland. Nineteen dogs had left complete CR and 10 dogs had right complete CR. All dogs had unilateral palpable cranial translation of the tibia relative to the femur on sedated exam, indicative of complete CR. Dogs had no evidence of cranial drawer or cranial tibial thrust in the contralateral stable CR stifle under sedation but did have radiographic evidence of joint effusion and/or OA, suggesting partial CR (Grade I sprain). However, under general anesthesia, four dogs were noted to have evidence of a Grade II sprain in the partial CR stifle. Two dogs had a subtle cranial drawer of the partial CR stifle with <3mm of translation when the stifle was placed in flexion but did not have cranial drawer when the stifle was in extension; neither of these dogs developed a complete CR in this stifle within the 12-month study period. A third dog had subtle mild cranial drawer with <3mm of translation of the partial CR stifle in both flexion and extension; this dog went on to develop a complete CR rupture in this stifle within the 12-month study period. A fourth dog had subtle cranial drawer with <3mm of tibial translation of the tibia and also had a <3mm of positive tibial thrust test in the partial CR stifle; this dog did not develop a complete CR rupture in this stifle within the 12-month study period.

All dogs returned for the 10-week recheck at 73±12 days after surgery (range: 59–124 days), at which time 2 dogs were noted to have developed mild instability of the PRP-collagen treated stifle with sedated examination, which was not present at diagnosis; both of these dogs developed complete CR within the study period. These dogs did not have Grade II sprain identified under general anesthesia at the time of diagnosis.

Twenty-seven dogs completed the study. During the 12-month study duration, 8 of 27 (29.6%) dogs experienced a complete CR of the PRP-collagen treated stifle at a median time of 214 days (range 89–285 days). Nineteen dogs returned for the 12-month recheck at 385±24 days after surgery (range: 349–432 days). None of these dogs had evidence of complete CR in the PRP-collagen treated stifle. Two dogs did not complete the study; one dog died 222 days after TPLO surgery and PRP-collagen treatment because of autoimmune disease, and one dog was lost to follow-up after the 10-week recheck and did not return for the 12-month recheck.

No significant correlations were found between time to, or occurrence of, complete CR in the PRP-collagen treated stifle with partial CR and age of diagnosis, gender, weight or body condition score (S2 Table).

### Platelet recovery and concentration

PRP was successfully isolated from whole blood in all dogs. Centrifugation of the 32mL of blood from each dog resulted in a mean of 3.6±0.23mL (range: 3.1–4.2) PRP. The mean percentage platelets recovered was 71.4±10.18% (range: 51.3–95.9). The mean platelet concentration factor was 6.4±0.82 (range: 4.32–7.86).

### Radiography, MR imaging and arthroscopy

**Fig 2:**
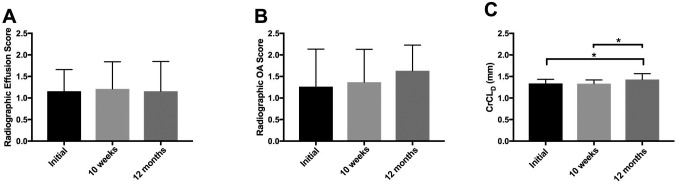
Changes in radiographic and morphometric variables in the PRP-collagen treated stifle over time. **(A,B)** Radiographic effusion and OA did not significantly differ between study time points. **(C)** CrCL_D_ significantly increased from both the time of diagnosis and the 10-week recheck to the 12-month recheck. **Abbreviations**: PRP, platelet rich plasma; CrCL_D_, radiographic length of the CrCL normalized to patellar length; OA, osteoarthritis. n = 19 dogs.

**Fig 3:**
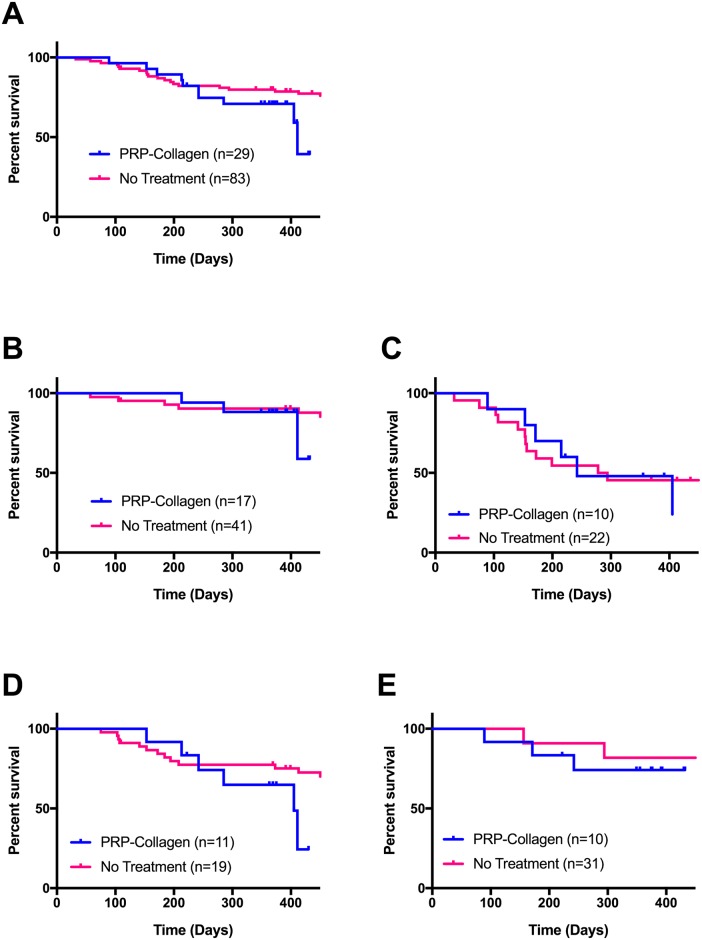
Partial CR stifle cranial cruciate ligament (CrCL) time to complete rupture in dogs with and without PRP-collagen treatment. Kaplan-Meier plots for CrCL time to rupture of partial CR stifles with and without PRP-collagen treatment [1]. Cases that developed a complete CR (Grade III ligament rupture) were censored for analysis. Arthroscopic examination and associated lavage, together with intra-articular PRP-collagen, did not significantly influence time to complete CR over the study period (Table 3). (A) Development of complete CR in all partial CR stifles. (B) Development of complete CR in stifles with Grade 1 radiographic effusion at diagnosis. (C) Development of complete CR in stifles with Grade 2 radiographic effusion at diagnosis. (D) Development of complete CR in stifles with Grade 1 radiographic OA at the time of diagnosis. (E) Development of complete CR in stifles with Grade 2 radiographic OA at the time of diagnosis. **Abbreviations**: PRP, platelet rich plasma; CR, cruciate rupture; CrCL, cranial cruciate ligament; OA, osteoarthritis; TPLO, tibial plateau leveling osteotomy.

Correlations between diagnostic variables and time to, or occurrence of, complete CR in the PRP-collagen treated partial CR stifles are summarized in Tables 1 and 2, respectively. Radiographs were obtained in all 29 dogs at the time of diagnosis and at the 10-week recheck and were obtained in 19 dogs at the 12-month recheck. The TPA of the complete CR stifle at diagnosis significantly correlated with time to complete CR in the PRP-collagen treated partial CR stifle (S_R_ = 0.86, *P*<0.01) (Table 1). Severity of radiographic synovial effusion in the partial CR stifle treated with PRP-collagen correlated with development of complete CR at 12 months (S_R_ = 0.41, *P*<0.05) (Table 2). CrCL_D_ of the PRP-collagen treated stifle was increased at the 12-month recheck compared to both initial and 10-week time points (*P*<0.05) (Fig 2). No other radiographic variables changed significantly over the study period. Time to complete CR in the PRP-treated partial CR stifles was not significantly different from the historic control of partial CR stifles (*P* = 0.26) [1] (Fig 3), nor were significant differences seen between these groups when dogs were stratified by severity of radiographic effusion or radiographic OA (Table 3, Fig 3).

**Table 1:** Correlation between time to complete CR in the PRP-collagen treated stifles and variables obtained at the time of diagnosis.

Variable	TPLO treated complete CR stifle	PRP-collagen treated partial CR stifle
**Radiographic and morphometric variables**		
Effusion	#	S_R_ = -0.57, *P* = 0.16
Osteophytosis	S_R_ = -0.06, *P* = 0.93	S_R_ = -0.26, *P* = 0.53
CrCL_D_	S_R_ = 0.58, *P* = 0.13	S_R_ = 0.64, *P* = 0.09
TPA	*S*_*R*_ = *0*.*86*, *P = 0*.*008*	S_R_ = 0.68, *P* = 0.07
**MR imaging quantification**		
CrCL FSE volume (mm^3^/mm)	S_R_ = -0.56, *P* = 0.21	S_R_ = 0.74, *P* = 0.07
CrCL VIPR volume (mm^3^/mm)	n/a	S_R_ = 0.30, *P* = 0.68
CrCL FSE Grayscale	S_R_ = 0.40, *P* = 0.38	S_R_ = -0.40, *P* = 0.38
CrCL VIPR Grayscale	n/a	S_R_ = -0.80, *P* = 0.13
CrCL T1 Enhance	n/a	S_R_ = -0.61, *P* = 0.16
CrCL Fiber Tearing	S_R_ = -0.04, *P* = 0.94	S_R_ = -0.77, *P* = 0.053
**Arthroscopy**		
Synovitis Score	S_R_ = -0.71, *P* = 0.054	S_R_ = -0.10, *P* = 0.82
Synovitis VAS	S_R_ = -0.24, *P* = 0.56	S_R_ = -0.45, *P* = 0.26
CrCL Fiber Damage VAS	n/a	S_R_ = -0.09, *P* = 0.83
**Biomarkers**		
Serum CRP	S_R_ = 0.14, *P* = 0.74	
Synovial CRP	S_R_ = 0.20, *P* = 0.63	S_R_ = -0.25, *P* = 0.55
Synovial:Serum CRP	S_R_ = -0.29, *P* = 0.49	S_R_ = -0.33, *P* = 0.41
TNCC	S_R_ = 0.31, *P* = 0.71	S_R_ = 0.57, *P* = 0.14
**Histology**		
Synovial Inflammation VAS	S_R_ = -0.06, *P* = 0.90	S_R_ = -0.37, *P* = 0.36
Synovitis Grade	S_R_ = -0.38, *P* = 0.39	S_R_ = -0.28, *P* = 0.52

n = 5–8 dogs. **Abbreviations**: PRP, platelet rich plasma; CrCL, cranial cruciate ligament; CrCL_D_, radiographic length of the CrCL normalized to patellar length; BCS, body condition score; FSE, 3D fast spin echo; VIPR, Vastly under-sampled Isotropic Projection; VAS, visual analog scale; CRP, C-reactive protein; TNCC, total nucleated cell count; n/a, not applicable.

^**#**^Synovial effusion in all TPLO-treated complete CR stifles was 2.

**Table 2:** Correlation between occurrence of complete CR at 12 months after diagnosis in PRP-collagen treated stifles and variables obtained at the time of diagnosis.

Variable	TPLO treated complete CR stifle	PRP-collagen treated partial CR stifle
**Radiographic and Morphometric variables**		
Effusion	S_R_ = 0.13, *P* = 0.52	*S*_*R*_ = *0*.*41*, *P = 0*.*035*
Osteophytosis	S_R_ = 0.10, *P* = 0.61	S_R_ = 0.12, *P* = 0.57
CrCL_D_	S_R_ = -0.16, *P* = 0.42	S_R_ = -0.31, *P* = 0.11
TPA	S_R_ = 0.13, *P* = 0.53	S_R_ = 0.19, *P* = 0.33
**MR imaging quantification**		
CrCL FSE volume (mm^3^/mm)	S_R_ = -0.18, *P* = 0.38	S_R_ = -0.23, *P* = 0.27
CrCL VIPR volume (mm^3^/mm)	n/a	S_R_ = -0.42, *P* = 0.08
CrCL FSE Grayscale	S_R_ = 0.10, *P* = 0.63	S_R_ = -0.10, *P* = 0.63
CrCL VIPR Grayscale	n/a	S_R_ = -0.04, *P* = 0.89
CrCL T1 Enhance	n/a	S_R_ = -0.03, *P* = 0.89
CrCL Fiber Tearing	S_R_ = -0.25, *P* = 0.21	S_R_ = 0.24, *P* = 0.23
**Arthroscopy**		
Synovitis Score	S_R_ = -0.31, *P* = 0.12	S_R_ = 0.08, *P* = 0.68
Synovitis VAS	S_R_ = -0.09, *P* = 0.66	S_R_ = 0.27, *P* = 0.18
CrCL Fiber Damage VAS	n/a	*S*_*R*_ = *0*.*39*, *P = 0*.*047*
**Biomarkers**		
Serum CRP	S_R_ = 0.03, *P* = 0.88	
Synovial CRP	S_R_ = -0.09, *P* = 0.64	S_R_ = -0.06, *P* = 0.79
Synovial:Serum CRP	S_R_ = -0.23, *P* = 0.25	S_R_ = -0.09, *P* = 0.67
TNCC	*S*_*R*_ = -*0*.*52*, *P = 0*.*007*	S_R_ = -0.18, *P* = 0.39
**Histology**		
Synovial Inflammation VAS	S_R_ = 0.25, *P* = 0.21	S_R_ = 0.04, *P* = 0.84
Synovitis Grade	S_R_ = 0.11, *P* = 0.57	S_R_ = 0.05, *P* = 0.79

n = 18–27 dogs. **Abbreviations**: PRP, platelet rich plasma; CrCL, cranial cruciate ligament; CrCL_D_, radiographic length of the CrCL normalized to patellar length; BCS, body condition score; FSE, 3D fast spin echo; VIPR, Vastly under-sampled Isotropic Projection; VAS, visual analog scale; CRP, C-reactive protein; TNCC, total nucleated cell count; n/a, not applicable.

**Table 3:** Relationship of radiographic synovial effusion and radiographic OA in partial CR stifles and rupture within 1 year of diagnosis.

Grade	Control Ruptured	Control Not Ruptured	PRP Ruptured	PRP Not Ruptured	Chi Square Statistic	Significance
**All Dogs**						
	17	66	8	19	0.971	0.324
**Radiographic Effusion**						
0	1	19	0	1	n/a	
1	4	37	3	14	0.705	0.401
2	12	10	5	4	0.003	0.959
**Radiographic Osteophytosis**						
0	0	1	1	4	n/a	
1	2	17	3	7	1.741	0.187
2	6	25	3	7	0.500	0.479
3	9	23	1	1	0.434	0.510

All dogs were diagnosed with unilateral complete CrCL rupture and contralateral partial CR rupture. Control dogs (n = 83) did not receive PRP-collagen treatment or other intervention (Chuang et al. 2014). PRP dogs (n = 27) received intra-articular PRP-collagen at the time of contralateral TPLO surgery.

No significant correlations were seen between MRI or arthroscopic variables and time to, or occurrence of, complete CR in the PRP-collagen treated stifle (Tables 1 and 2). However, CrCL fiber tearing and CrCL FSE volume of the PRP-collagen treated stifle at diagnosis were both weakly correlated with time to rupture (S_R_ = -0.77, *P* = 0.05; S_R_ = 0.74, *P* = 0.07, respectively) (Table 1). PRP-collagen treated stifle CrCL FSE volume and CrCL VIPR volume significantly decreased (*P*<0.01) from the time of diagnosis to the 12-month recheck (Table 4). No other MR imaging variables changed significantly over the study period.

**Table 4:** MRI variables from PRP-collagen treated stifles with partial cruciate ligament rupture.

Variable	n	Time of diagnosis	12-month recheck	*P*-value
CrCL FSE volume (mm^3^/mm)	17	24.35±6.63	19.68±4.4	*0*.*002*
CrCL VIPR volume (mm^3^/mm)	10	23.57±6.12	16.43±7.39	*0*.*004*
CrCL FSE Grayscale^a^	17	0.84±0.27	0.99±0.40	0.38
CrCL VIPR Grayscale^a^	10	0.89±0.26	1.08±0.37	0.28
CrCL T1 Enhance	17	0.87 (0.50–3.53)	1.18±0.39	0.21
CrCL Fiber Loss	17	47.5 (1–86)	48.29±7.58	0.14

**Abbreviations**: CrCL, cranial cruciate ligament; FSE, fast spin echo; VIPR, Vastly under-sampled Isotropic Projection.

^a^Ligament grayscale value was normalized to the cranial tibial muscle grayscale value.

### Inflammation in synovium, synovial fluid and serum

**Fig 4:**
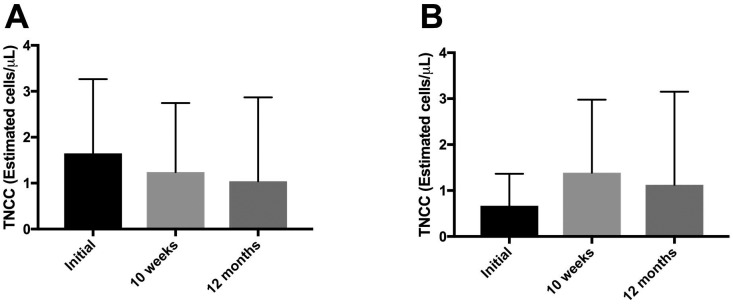
Changes in estimated total nucleated cell counts over time. Estimated TNCC from the synovium of **(A)** PRP-collagen treated partial CR stifles and **(B)** TPLO-treated complete CR stifles did not significantly change over the study period. **Abbreviations**: TNCC, total nucleated cell count; CR, cruciate rupture; PRP, platelet rich plasma; TPLO, tibial plateau leveling osteotomy. n = 19 dogs.

**Fig 5:**
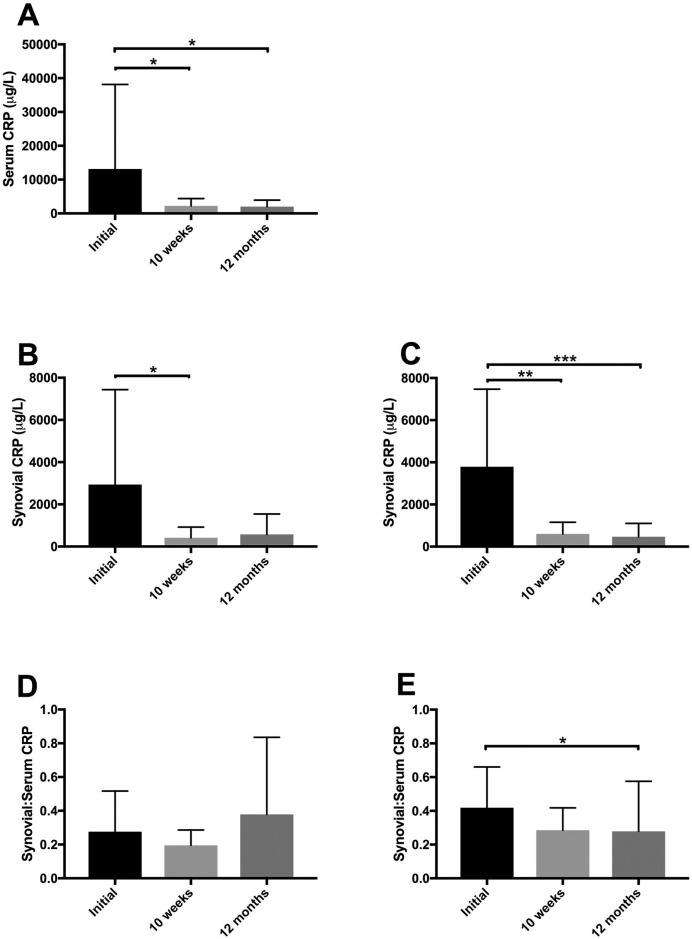
Changes in C-reactive protein over time. **(A)** Serum CRP concentrations significantly decreased at the 10-week and 12-month rechecks compared to initial concentrations obtained at the time of diagnosis. **(B)** The PRP-collagen treated stifle synovial CRP was significantly decreased at the 10-week recheck and trended towards being significantly decreased at the 12-month recheck. **(C)** The TPLO treated stifle synovial CRP was significantly decreased at the 10-week and 12-month rechecks compared to initial levels obtained at the time of diagnosis. **(D)** The PRP-collagen treated stifle synovial:serum CRP ratio did not significantly change between time points, although **(E)** in the TPLO treated stifle, synovial:serum CRP significantly decreased between the time of diagnosis and the 12-month recheck. **Abbreviations**: CR, cruciate rupture; PRP, platelet rich plasma; CRP, C-reactive protein; TPLO, tibial plateau leveling osteotomy. *P<0.05, **P<0.01, ***P< 0.001. n = 19 dogs.

Correlation between diagnostic variables and time to, or occurrence of, complete CrCL rupture in the PRP-collagen treated stifle are summarized in Tables 1 and 2, respectively. Estimated TNCC of the TPLO treated stifle at diagnosis and occurrence of complete CR in the PRP-collagen treated partial CR stifle were significantly and negatively correlated (S_R_ = -0.52, *P*<0.01). Estimated TNCCs levels did not change over the study period in either the PRP-collagen treated partial CR stifle or the TPLO-treated complete CR stifle (Fig 4). No other significant correlations were seen between measures of inflammation and time to, or occurrence of, complete CR in the PRP-collagen treated stifle. Serum CRP decreased at the 10-week and 12-month rechecks (*P*<0.05) compared with initial levels obtained at the time of diagnosis (Fig 5A). Compared with time of diagnosis, synovial CRP in the PRP-collagen treated stifle was also decreased at the 10-week recheck (*P*<0.05) (Fig 5B), and the 12-month recheck (*P* = 0.16, β = 0.6). Synovial CRP in the TPLO-treated complete CR stifle was decreased at the 10-week and 12-month rechecks (*P*<0.01, *P*<0.001, respectively) compared with initial levels obtained at the time of diagnosis (Fig 5C). Synovial:serum CRP in the TPLO-treated stifle was decreased at the 12-month recheck (*P*<0.05) compared with the time of diagnosis (Fig 5E).

### Development of complete CR in PRP-collagen treated partial CR stifles

Cox regression analysis was used to develop a predictive model for time to complete CR over the 12-month study period in the PRP-collagen treated partial CR stifles. The following variables were fitted to the model, based on univariate regression: PRP-collagen treated stifle Arthroscopic Fiber Damage VAS, PRP-collagen treated stifle Radiographic Effusion, PRP-collagen treated stifle TPA, PRP-collagen treated stifle CrCL FSE volume, and PRP-collagen treated stifle TNCC. This model resulted in a likelihood ratio of 19.5 (*P* = 0.0015) for time to complete CR in a PRP-collagen treated stifle joint within 12-months of diagnosis. A second model was also evaluated, using only a subset of radiographic measures taken from the PRP-collagen treated stifle at the time of diagnosis. The following variables, obtained from the PRP-collagen treated stifle at the time of diagnosis, were fitted to the model based on univariate analysis: radiographic effusion grade, radiographic OA grade, and CrCL_D_. This model resulted in a likelihood ratio of 7.61 (*P* = 0.055) for time to complete CR in the PRP-collagen treated stifle joint within 12 months of diagnosis.

## Discussion

Canine CR is progressive, complex disease with genetic and environmental risk. The frequency of contralateral rupture in dogs with unilateral complete CR ranges from 22–54% within one year of diagnosis [35–39]. Development of a second contralateral CR is related to the presence and severity of stifle synovitis [12] and radiographic severity of stifle synovial effusion and osteophytosis in the contralateral stable stifle at diagnosis [1]. In partial CR stifles, CrCL MR signal intensity is correlated with histologic synovitis, as is radiographic OA and arthroscopically assessed CrCL fiber damage [25]. The success of regenerative medicine treatments, such as PRP, to modify the CR disease process is little studied in dogs. The present study aimed to evaluate the use of an intra-articular PRP-collagen treatment as a disease-modifying therapy through analysis of arthroscopic findings, histologic assessment, radiographic findings, inflammatory markers and MR imaging changes over a 12-month study period, and to evaluate whether treatment had anti-inflammatory effects or lengthened time to complete CR. The outcome measures used in this work are as previously described [25]. Our results suggest that treatment of partial CR with a single application of a PRP-collagen hydrogel does not have a significant sparing effect on risk of disease progression to complete CR. In the PRP-collagen treated stifle, a number of variables obtained at the time of diagnosis correlated with risk of CR rupture by 12 months, including increased radiographic effusion, greater arthroscopic CrCL fiber damage, and loss of ligament volume measured by MR imaging using the VIPR sequence.

The signalment of dogs enrolled in this study is typical of the CR condition. Disease risk is breed-associated, with some breeds such as the Newfoundland and Rottweiler having high disease risk and others, such as the Greyhound, being protected [40]. All dogs enrolled in this study, and those used as historic controls, had evidence of a Grade III CrCL sprain in one stifle, which was treated with a TPLO, and a Grade I-II sprain in the contralateral stifle, for which the dogs in the present study were treated with a single treatment of intra-articular PRP-collagen. Grade of cruciate ligament sprain in the PRP-collagen treated stifle at diagnosis was based on careful palpation under general anesthesia. Four dogs were diagnosed with Grade II sprains, although only one went on to develop a complete CR during the study period. Interestingly, two dogs that did not have Grade II sprain at diagnosis developed mild instability detectable under sedation by the 10-week recheck; both of these dogs went on to develop a complete Grade III sprain within the study period. Risk factors for development of bilateral CR include breed, age and body weight [38,40,41]. Significant correlations between these factors and development of a second cruciate ligament rupture were not identified, which is likely a reflection of purposeful selection bias, as only those dogs with evidence of bilateral disease were eligible for enrollment.

The relationship between activity after unilateral stifle stabilization and progression of CR in a contralateral partial CR stifle is not known. In the present study, owners were provided with specific post-operative instructions regarding their dog’s activities during covalence from TPLO surgery. However, a limitation of studies involving client-owned animals includes an inability to strictly control environmental factors such as activity level.

The present study did not show significant reduction in progression to complete CR within 12 months of a single application of PRP-collagen. However, close scrutiny of Kaplan Meier curves illustrating time to rupture in PRP-collagen treated dogs verses a control population (Fig 3) provides some evidence of an early protective treatment effect. Furthermore, synovial CRP in the PRP-collagen treated stifle was decreased at 10-weeks, suggesting that the PRP-collagen treatment was anti-inflammatory. There was also a lack of progression in radiographic effusion and radiographic OA scores in the PRP-collagen treated stifles over the study period. Taken together, these findings suggest that PRP-collagen treatment has an effect on the inflammatory environment within stifles with partial CR and may be more effective with repeated applications.

The correlation between increased radiographic effusion in stable stifles and the development of complete CR by 12 months after diagnosis has been shown previously [1,3]. However, we did not find a correlation with severity of osteophytosis, as was the case in earlier work. Interestingly, we also did not find a correlation between occurrence of CR rupture in PRP collagen treated stable stifles and arthroscopic synovitis. Arthroscopic evaluation of the stifle is considered the gold standard method for assessment of cruciate ligaments, articular cartilage and synovitis [5,42]. Synovitis is an important contributor to development of CR [5]. The lack of correlation between the degree of synovitis at diagnosis and development of CR over the 12-month study period may be a reflection of PRP-collagen therapy, as decreases in synovial CRP levels in PRP-collagen treated joints were seen within 10 weeks of treatment, suggesting an anti-inflammatory effect. It is also possible that degree of synovitis in a diseased joint is dynamic, and evaluation at a single time point in the course of disease progression is not fully reflective of the risk of disease progression. The negative correlation between TNCC in the TPLO-treated stifle and development of CR over the study period in the contralateral PRP-collagen treated stifle was unexpected. We can only surmise that synovial macrophages may contribute to ligament homeostasis, although whether PRP-collagen treatment influenced this potential relationship is unclear. However, once CrCL fiber damage as developed as a result of the mechanical and inflammatory environment with the stifle, this significantly influences development of complete CR at 12 months.

The inverse correlation between development of CR within 12 months of diagnosis and CrCL ligament volume determined using MR imaging with the VIPR sequence is of particular interest. Previous work evaluating correlations between arthroscopic CrCL ligament damage and volume measurements obtained through MR imaging did not show these variables to be correlated [25]. Ligament volume determined through MR imaging and arthroscopic estimates of fiber damage each measure different features of the CrCL ligament. Ligament volume may be influenced by pathologies such as edema and scar formation that could be better identified through MR imaging than through arthroscopic examination. Ligament volume on MRI is a clinically-relevant marker of mechanical properties [29] Collectively, this suggests that CrCL volume measurements obtained through MR imaging are valuable non-invasive methods to estimate the mechanical status of the CrCL in stable stifles with partial CR.

We considered both time to development of complete CR and occurrence of complete CR within 12 months of diagnosis as outcomes. Each of these outcomes reflects different aspects of the CR disease condition. Time to development of CR is perhaps more reflective of the rate of progression of ligament fiber tearing. Correlations with development of complete CR at 12-months after treatment are perhaps less reflective of the rate of disease progression but allows for comparison between dogs that develop a threshold level of damage in the ligament within a specific time period and dogs that do not. It is important to note that in the present study, correlations with development of complete CR at 12 months only included the 8 dogs in which this was identified during the study period.

While no significant correlations were seen between time to CR in PRP-collagen treated partial CR stifles within the study period and the diagnostic variables measured, synovitis in the TPLO-treated complete CR stifle was inversely correlated with time to contralateral rupture, a similar finding to earlier work [12]. Unexpectedly, we found that TPA of the TPLO- and PRP-collagen treated stifles at diagnosis had a positive correlation with time to CR rupture. TPA has been investigated as a risk factor for the development of CR with conflicting results [43–45], although the effect of TPA on progression of disease over time is not known. Our results suggest that an increasing TPA does not promote rate of disease progression to result in a shorter time to development of complete CR, although this result should be considered with caution due to the low number of dogs in the time to rupture analysis.

Radiographic effusion and osteophytosis were not found to be correlated with time to rupture, although time to rupture was weakly correlated with effusion (S_R_ = -0.57, *P* = 0.16), fitting with the general observation that radiographic synovial effusion is clinically relevant marker for disease progression. However, we found that increased CrCL fiber tearing, determined using MR imaging, correlated with shorter times to development of complete CR. This finding was expected. Interestingly, the degree of CrCL fiber damage identified arthroscopically at the time of diagnosis in the PRP-collagen treated partial CR stifle did not correlate well with time to development of CR, suggesting that MR imaging may provide better overall assessment of the mechanical integrity of the CrCL. Without evaluations at intermediate time points between diagnosis and development of complete CR, further conclusions cannot be drawn. The use of the 3D FSE Cube and VIPR-aTR sequences for evaluation of CrCL structural properties in the dog has been described [25,29], and CrCL fiber tearing evaluated through MR imaging has been shown to be a sensitive and specific means to evaluate CrCL damage in stifles with partial CR rupture, when arthroscopic assessment is used as a gold standard [30]. The contrast between the two methods of determining CR fiber tearing or damage, either through arthroscopy or MR imaging, and the correlations with time to CR development is interesting, and merits further investigation. Future work evaluating the natural history of CrCL damage development through MR imaging would help to clarify relationships between MR measured CrCL volume and grayscale values and development of progressive fiber rupture over time.

Radiographic and MR imaging of the PRP-collagen treated stifles was repeated at 12 months in dogs that did not develop complete CR. Radiographic effusion and OA grades did not significantly change over the treatment period in the PRP-collagen treated stifles. The lack of a change in radiographic OA was expected, as it is unlikely that established OA would regress even in the face of an anti-inflammatory treatment. The lack of a change in radiographic effusion is perhaps an indicator that the single treatment with PRP-collagen did not fully attenuate the inflammatory environment within the partial CR stifles, even though a significant decrease in synovial CRP was found in these joints. Interestingly, an increase was noted in the CrCL_D_ of the PRP-collagen treated partial CR stifles at 12-months, compared to both the time of diagnosis and the 10-week recheck, indicating that progressive fiber rupture likely occurred over the 12-month study period. The use of CrCL_D_ as a means to track progressive rupture in partial CR stifles over time is worthy of further investigation.

We also found that CrCL volume measured on MR images from both the 3D FSE Cube and VIPR-aTR sequences decreased significantly over the study period. Higher grayscale values from both sequences were also identified at 12 months. Together, these results suggest that the CrCL in the PRP-collagen treated stifles may have undergone some degree of healing with reduced tissue edema over time and an associated reduction in ligament volume. Loss of volume could also be interpreted as fiber loss in the ligament matrix, but severity of fiber loss evaluated using MR imaging at 12 months was similar to that at diagnosis.

CRP is an acute phase protein that is elevated with development of inflammation. CRP may be a marker for disease progression in osteoarthritis in people and dogs [25,46]. Serum and synovial CRP concentrations, obtained at diagnosis from both TPLO- and PRP-collagen treated stifles, did not correlate with disease progression in PRP-collagen treated stifles. Synovial CRP in PRP-collagen treated stifles was decreased at 10-weeks compared to time of diagnosis, but not at the 12-month recheck. This early decrease may be a consequence of the PRP-collagen treatment, although further study with dogs partial CR that were not treated with PRP-collagen would be needed to fully define this relationship. Synovial CRP in the TPLO-treated stifle was also decreased after surgery, suggesting that joint stabilization provided by the TPLO surgery helps to reduce the inflammatory environment within the stifle.

We used Cox regression analysis to build a predictive model for development of CR in PRP-collagen treated partial CR stifles, taking into account both occurrence of rupture and time to rupture within the 12-month study period. A model that includes a combination of arthroscopic, radiographic, morphometric, MR imaging and inflammatory markers provided significant prediction with a likelihood ratio of 19.5 for the development of complete CR. CR is a complex disease [2]. We found that multiple modalities were needed for robust prediction. To evaluate the use of measures that can be more easily obtained at the time of diagnosis, we also studied a model with a subset of radiographic measures, including effusion grade, OA grade and CrCL_D_; this model approached significance, and resulted in a likelihood ratio of 7.6 for development of CR. The effect of PRP-collagen treatment on these models cannot be determined in the present study. Further work validating the accuracy of both these models would best be undertaken in separate groups of client-owned dogs with partial CR with and without PRP-collagen treatment.

This study has several limitations. Use of client owned animals with a naturally occurring disease can be considered a limitation, although it is also a strength; this model has been developed as a means of evaluating disease-modifying treatment for canine CR [25]. We analyzed partial CR dogs that were treated with single application of intra-articular PRP-collagen and did not have a separate control group of dogs that did not receive the PRP-collagen treatment for ethical reasons. Instead, we used a historic control group for analysis of time to rupture data [1]. This study would benefit from a longer follow-up time period, as the median time to contralateral CrCL rupture in dogs with unilateral complete CR is 2.59 years [7]. The attending clinician on each case was responsible for evaluation of arthroscopic and clinical features of dogs at the time of diagnosis, and, therefore, variation in arthroscopic scoring and assessment of subtle differences in joint instability at diagnosis may have influenced our results. Determination of Grade I versus II sprains was undertaken by 1 of 3 attending board-certified veterinary surgeons. It is possible that interobserver variability may have resulted in inconsistent grade assignments. 3.0T MR imaging was obtained in 28 of the 29 dogs. The first dog enrolled in the study had MR imaging with a 1.5T machine, but the images obtained were deemed inadequate. The VIPR-aTR sequence was not obtained in all dogs, due to limited availability of this experimental sequence.

In conclusion, we examined use of a single application of a PRP-collagen hydrogel in partial stable CR joints using client-owned dogs with a unilateral complete CR and contralateral partial CR. We chose diagnostic variables based on previous work that evaluated outcome measures for this model [25]. Radiographic effusion, arthroscopic evaluation of CrCL damage, and MR assessment of ligament fiber tearing in partial CR stifles were significantly correlated with progression to complete CR over a 12-month follow-up period. A single treatment of partial CR with PRP-collagen did not significantly alter progression of disease in this study. Further work with this clinical model is needed to evaluate use of repeated intra-articular treatment with PRP-collagen or other regenerative medicine treatments. Finally, we found that inclusion of diagnostic variables from multiple modalities resulted in the best predictive model for development of complete CR in PRP-collagen treated partial CR stifles. Collectively, this work indicates that a single application of PRP-collagen in partial CR stifles of client owned dogs is not an effective disease-modifying therapy, although further work is needed.

## Supporting information

S1 Table Summary of parameter collection. (DOCX)

S2 Table Correlation between examination parameters and time to, or occurrence of, complete CR of the PRP-collagen treated stifle. (DOCX)

S3 Table Univariate Cox regression results. (DOCX)

S1 File Study data is provided in the supporting excel file “S1 File” data. (XLSX)
