# Supplementary material for: Use of a platelet-rich plasma-collagen scaffold as a bioenhanced repair treatment for management of partial cruciate ligament rupture in dogs
Source: PLoS One. 2018 Jun 19;13(6):e0197204. doi: 10.1371/journal.pone.0197204 (PMC6008044; doi:10.1371/journal.pone.0197204)
Supplement: S1 Table — (DOCX) [file pone.0197204.s001.docx]

**S1 Table.** Summary of parameter collection

|  | **TPLO treated complete CR stifle** | | | **PRP-collagen treated partial CR**  **stifle** | | |
| --- | --- | --- | --- | --- | --- | --- |
| Variable | Time of Diagnosis | 10-week Recheck | 12-month recheck* | Time of Diagnosis | 10-week Recheck | 12-month recheck* |
| **Signalment and Exam** | | | | | | |
| Age, Weight, BCS, Gender | n=29 |  |  | n=29 |  |  |
| **Radiographic and Morphometric variables** | | | | | | |
| Synovial Effusion | n=29 | n=29 | n=19 | n=29 | n=29 | n=19 |
| Osteophytosis | n=29 | n=29 | n=19 | n=29 | n=29 | n=19 |
| CrCL_D_ | n=29 | n=29 | n=19 | n=29 | n=29 | n=19 |
| TPA | n=29 | n=29 | n=19 | n=29 | n=29 | n=19 |
| **MR Imaging Quantification** | | | | | | |
| CrCL FSE Volume | n=28 |  |  | n=28 |  | n=17 |
| CrCL VIPR Volume |  |  |  | n=19 |  | n=16 |
| CrCL FSE Grayscale | n=28 |  |  | n=28 |  | n=17 |
| CrCL VIPR Grayscale |  |  |  | n=19 |  | n=16 |
| CrCL T1 Enhance |  |  |  | n=28 |  | n=17 |
| CrCL Fiber Tearing | n=28 |  |  | n=28 |  | n=17 |
| **Arthroscopy** | | | | | | |
| Synovitis Score | n=29 |  |  | n=29 |  |  |
| Synovitis VAS | n=29 |  |  | n=29 |  |  |
| CrCL Fiber Damage VAS |  |  |  | n=29 |  |  |
| **Biomarkers** | | | | | | |
| Serum CRP | n=29 | n=29 | n=19 | n=29 | n=29 | n=19 |
| Synovial CRP | n=29 | n=28 | n=19 | n=28 | n=28 | n=17 |
| Synovial:Serum CRP | n=29 | n=28 | n=19 | n=28 | n=28 | n=17 |
| TNCC | n=27 | n=28 | n=19 | n=26 | n=28 | n=19 |
| **Histology** | | | | | | |
| Synovial Inflammation VAS | n=29 |  |  | n=29 |  |  |
| Synovitis Grade | n=29 |  |  | n=29 |  |  |

**Note**: *Only obtained in stifles that did not rupture by the 12-month recheck. **Abbreviations**: TPLO, tibial plateau leveling osteotomy; PRP, platelet rich plasma; CrCL, cranial cruciate ligament; CrCL_D_, radiographic length of the CrCL normalized to patellar length; BCS, body condition score; FSE, 3D fast spin echo; VIPR, Vastly under-sampled Isotropic Projection; VAS, visual analog scale
