# Supplementary material for: Use of a platelet-rich plasma-collagen scaffold as a bioenhanced repair treatment for management of partial cruciate ligament rupture in dogs
Source: PLoS One. 2018 Jun 19;13(6):e0197204. doi: 10.1371/journal.pone.0197204 (PMC6008044; doi:10.1371/journal.pone.0197204)
Supplement: S2 Table — (DOCX) [file pone.0197204.s002.docx]

**S2 Table.** Correlation between examination parameters and time to, or occurrence of, complete CR of the PRP-collagen treated stifle

| **Parameter** | **Time to Rupture** | **Occurrence of Rupture** |
| --- | --- | --- |
| Age at Diagnosis | S_R_ = 0.32, *P* = 0.43 | S_R_ = -0.07, *P* = 0.72 |
| Gender | S_R_ = 0.11, *P* = 0.84 | S_R_ = 0.18, *P* = 0.35 |
| Weight | S_R_ = -0.19, *P* = 0.65 | S_R_ = -0.27, *P* = 0.17 |
| Body Condition Score | S_R_ = -0.54, *P* = 0.16 | S_R_ = -0.16, *P* = 0.41 |
