# Supplementary material for: Use of a platelet-rich plasma-collagen scaffold as a bioenhanced repair treatment for management of partial cruciate ligament rupture in dogs
Source: PLoS One. 2018 Jun 19;13(6):e0197204. doi: 10.1371/journal.pone.0197204 (PMC6008044; doi:10.1371/journal.pone.0197204)
Supplement: S3 Table — (DOCX) [file pone.0197204.s003.docx]

| **Parameter** | ***P*-value** | | |
| --- | --- | --- | --- |
| **Signalment** | | | |
| Age at Diagnosis | 0.67 | | |
| Gender | 0.74 | | |
| Weight | 0.25 | | |
| Body Condition Score | 0.55 | | |
|  | **Complete CR Stifle** | **PRP Treated Partial CR Stifle** |  |
| **Radiographic and Morphometric variables** | | | |
| Effusion | 1 | 0.02 |  |
| Osteophytosis | 0.41 | 0.71 |  |
| CrCL_D_ | 0.58 | 0.4 |  |
| TPA | 0.28 | 0.14 |  |
| **MR imaging quantification** | | | |
| CrCL FSE volume (mm^3^/mm) | 0.84 | 0.2 |  |
| CrCL VIPR volume (mm^3^/mm) | n/a | 0.34 |  |
| CrCL FSE Grayscale | 0.61 | 0.6 |  |
| CrCL VIPR Grayscale | n/a | 0.97 |  |
| CrCL T1 Enhance | n/a | 0.44 |  |
| CrCL Fiber Tearing | 0.53 | 0.39 |  |
| **Arthroscopy** | | | |
| Synovitis Score | 0.77 | 0.25 |  |
| Synovitis VAS | 0.51 | 0.028 |  |
| CrCL Fiber Damage VAS | n/a | 0.0032 |  |
| **Biomarkers** | | | |
| Serum CRP | 0.73 | | |
| Synovial CRP | 0.26 | 0.43 |  |
| Synovial:Serum CRP | 0.15 | 0.45 |  |
| TNCC | 0.045 | 0.19 |  |
| **Histology** | | | |
| Synovial Inflammation VAS | 0.96 | 0.98 |  |
| Synovitis Grade | 0.91 | 0.91 |  |

**S3 Table.** Univariate Cox Regression Results
